# Molecular and Serological Investigation of Equine Herpesvirus Type 1 (EHV-1) and Type 4 (EHV-4) in Horses In Ibagué, Tolima

**DOI:** 10.1155/vmi/1661949

**Published:** 2025-01-29

**Authors:** Julieth Michel Petano-Duque, Edwin Urueña-Martinez, Laura Liliana Cabezas-Callejas, Jorge Perilla-Amaya, Valentina Rueda-García, Iang Schroniltgen Rondón-Barragán, Ricaurte Lopera-Vásquez

**Affiliations:** ^1^Research Group in Immunobiology and Pathogenesis, Laboratory of Immunology and Molecular Biology, Faculty of Veterinary Medicine and Zootechnics, Universidad del Tolima, Santa Helena Highs, Ibagué-Tolima 730006299, Colombia; ^2^Faculty of Veterinary Medicine and Zootechnics, Universidad Cooperativa de Colombia, Ibagué-Tolima, Colombia; ^3^Poultry Research Group, Laboratory of Immunology and Molecular Biology, Faculty of Veterinary Medicine and Zootechnics, Universidad del Tolima, Santa Helena Highs, Ibagué-Tolima 730006299, Colombia; ^4^Impronta Research Group, Universidad Cooperativa de Colombia, Ibagué-Tolima, Colombia

**Keywords:** EHV-1, EHV-4, ELISA, hnPCR, horse, PCR, serology

## Abstract

EHV-1 is one of the most prevalent viral pathogens in horses; however, its prevalence is not well defined by cross-reactions of serological tests. Thus, this study aims to determine EHV-1/-4 prevalence in Colombian creole horses from Ibagué, Tolima, using molecular techniques, as well as to determine possible risk factors associated with viral infection. A cross-sectional study was carried out in 110 equines blood, serum, and semen samples from Ibagué, Tolima. Antibodies against EHV-1/-4 were determined through indirect ELISA. EHV-1 was detected by amplifying the glycoprotein H gene through heminested PCR, and risk factors were calculated with chi-square, odds ratio, and fisher test. EHV-1/-4 antibodies were found in 11.8% of the horses. Moreover, the presence of EHV-1 and EHV-4 was determined in blood, serum, and semen of healthy clinical equines, with a prevalence infection in horses of 43.36% for EHV-1 and 2.7% for EHV-4. Sex, age, births number, abortions, natural breeding, and artificial insemination did not show significance as risk factors. This study determined a high prevalence of EHV-1 and low prevalence of EHV-4 in horses from Ibagué, Tolima. Furthermore, the possibility of finding the infectious agent in blood, serum, and semen was demonstrated. We recommend the use of blood and serum samples for virus detection and the development of new studies to assess EHV-1 infection feasibility in venereal transmissions.

## 1. Introduction

Among the emerging and constant threats to the equine industry worldwide, infection with equine herpesvirus type 1 (EHV-1), also known as equine abortion virus, and EHV-4 causes enormous economic losses [1–3]. These double-stranded virus belonging to the family Herpesviridae, subfamily Alphaherpesvirinae, and genus *Varicellovirus* are two of the most important and prevalent horse viral pathogens [1]. EHV-1/-4 can be latent in sensory neurons by intra-axonal transmission or host lymphocytes and reactivate to produce a lytic infection in internal organs endothelial cells and respiratory tract, or in mononuclear cells of lymphoid organs and peripheral blood, with a short and efficient replication cycle, propagating to cause cytopathic effects and the development of intracellular eosinophilic inclusion bodies [1]. EHV-1 mainly causes mild respiratory diseases and sporadic fever, but viral dissemination to distant organs allows more serious sequelae development such as abortions, perineal and neonatal mortality, and neurological diseases (equine herpes myeloencephalopathy) [4, 5]. In contrast, EHV-4 infection is highly limited to respiratory tract, with occasional neurological disorders and abortion [6, 7], and viremia is considered infrequent [3].

EHV-1 infection is highly contagious and can be acquired through contact with infectious materials such as water, saliva, nasal secretion, and aerosols, or contact with infected horses [2, 8]. Several methods for EHV-1 isolation and detection have been reported such as direct immunofluorescence, immunohistochemistry, and polymerase chain reaction (PCR), the latter being the fastest, reliable, and sensitive [9, 10]. Furthermore, thehigh cross-reactivity and variable latency periods observed in EHV-1 and EHV-4 serological tests complicate epidemiological analysis [1, 10].

In Colombia, EHV-1 and EHV-4 have been reported; however, systematic studies to determine the epidemiology of the virus are few [9, 11]. Due to this, the aim of this study is to determine EHV-1/-4 prevalence inhorses from Ibagué, Tolima, using serological and molecular techniques, as well as to assess the risk factors associated with the presence of the virus.

## 2. Materials and Methods

### 2.1. Ethical Statement

All the experimental procedures followed the guidelines for animal care and use in research and teaching based on Law 84/1989 and Resolution 8430/1993 and the guidelines of the bioethics committee of the Universidad Cooperativa de Colombia Act No. 002 from March 18th of 2020, bioethics concept No. 003-2020.

### 2.2. Sample Size and Collection

Statistical sample size was determined based on Thrusfield and Christley [12] with a confidence level of 99%, an accepted absolute error of 10%, an expected prevalence of 20% [9], and an equine population of 5.632 horses in Ibagué, Tolima [13], in a simple random sampling design. According to the formula, at least 106 samples should be collected.

In present study, 226 samples were collected from 110 clinically healthy horses from Ibagué farms during 2021-2022. Whole blood extraction was performed by peripheral venipuncture in the equine jugular vein, 110 whole blood samples were collected in tubes with ethylenediaminetetraacetic acid (EDTA) and tubes without anticoagulant and centrifuged at 1500 rpm for 10 min after clotting. In addition, 42 semen samples were collected by using an artificial vagina attached to a collection tube through sexual stimulation of a behaviourally oestrous mare and a ghost-mounted stallion. All samples were stored at −20°C until further processing.

### 2.3. Epidemiological Survey

A survey was conducted to determine the risk factors associated with EHV-1 and EHV-4 infection in equine populations, data included sex (mare and stallion) and age (young, ≤ 4 years; adult, 5 ≤ 9 years; old, ≥ 10 years [14], Moreover, number of births, foals born alive, foals born dead, and misbirths were collected for detection of associations between reproductive parameters and EHV-1-positive mares. Breeding techniques (natural mount, NM; and semen collection, SC) were assessed for EHV-1 venereal transmission in stallions.

### 2.4. Serological Detection

Enzyme-linked immunosorbent assays (ELISAs) were developed to detect antibodies against EHV-1/-4 in 110 serum samples per duplicate, using INgezim Rinoneumonitis kit (14.HVE.K.1/2 Ingenasa, Madrid, Spain) and following manufacturer's guidelines. The calculation of the positivity index (M/P) was carried out from the arithmetic mean of the two absorbance at 405 nm obtained for each sample. Those samples whose M/P index was greater than or equal to 0,3 were considered positive, and those in which it was less than 0,3 were considered negative.

### 2.5. DNA Extraction and Quality

Genomic DNA (gDNA) was extracted from whole blood, serum, and semen samples using phenol–chloroform isoamyl–alcohol (25:24:1) with a modification in cell lysis of semen samples [15]. Integrity and quality of the gDNA were verified by gel electrophoresis, NanoDrop One spectrophotometer (ThermoFisher, USA), and amplification of actin beta (*ACTB*) gene (Table 1) by PCR.

### 2.6. PCR and Heminested PCR (hnPCR)

Molecular detection of EHV-1 in horses was carried out by amplification of glycoprotein H gene (*gH*) and the open reading frame 30 (ORF30) and glycoprotein B (*gB*) and the ORF68 for EHV-4, through hnPCR using specific primers (Table 1).

PCR was performed following the procedures of Giraldo-Martinez et al. [18]. Briefly, a ProFlex PCRSystem thermocycler (Applied Biosystems, USA) was used for the amplificationreaction with a final reaction volume of 25 μL. This volume included 15.875 μL distilled-deionized water (ddw), 5 μL of5x colorless buffer, 1 μL of 1.5 mM dNTPs, 1 μL of each primer (forward andreverse; 10 pmol/μL), 0.125 μL GoTaq Flexi DNA polymerase (Promega, Madison,WI, USA), and 1 μL of template DNA. Amplification steps consisted of initial denaturation cycle at 95°C for 3 min, followed by 35 cycles of denaturation at 95°Cfor 30 s, annealing at 55°C for 30 s, extension at 72°C for 30 s, and final extension at 72°C for 5 min. EHV-1 positive control corresponds to viral DNA extracted from EHV-infected cell culture facilitated by Professor Agustin Gongora (Unillanos). ddw was used as negative control.

PCR products were revealed by 2% agarose gel electrophoresis, stained with Hydra green (ACTGene, USA), for 40 min at 100 V in the myGel mini electrophoresis chamber (ACCURIS, USA) and visualized under ultraviolet light using an ENDURO GDS gel documentation system (LabNet Intl, USA). Amplicons of ORFs were purified and sequenced by Sanger method (Macrogen Inc, South Korea).

### 2.7. Statistical Analysis

A database was created from the information collected in the clinical examinations and the epidemiological survey in the EpiInfo software (CDC, USA). Frequency tables were constructed, and relationship strength was determined by calculating the odds ratio (OR) and established through the Chi-square and Fisher tests. Values of *p* < 0.05 were considered statistically significant.

## 3. Results

### 3.1. Horse Population Characterization

From 110 horses, in total 68 were mares (61.81%) and 42 stallions (38.18%), 20 were young (18.18%), 67 adult (60.9%), and 23 old (20.9%). Regarding to mares, 32 have never been pregnant (47.05%), and 42 (61.76%) have a record between 1 and 6 births (*n = *21, 1; *n = *12, 2; *n = *4, 3; *n = *3, 4; *n = *2, 6) with a total of foals born alive between 1 and 6 (*n = *23, 1; *n = *12, 2; *n = *2, 3; *n = *3, 4; *n = *2, 6), and 1 (*n = *5) or 2 (*n = *1) abortions (9.6%). In the case of stallions, 3 (7.14%) have had natural breeding, and artificial inseminations have been practiced in 39 (92.3%).

### 3.2. Serological and Molecular Diagnosis

**Figure 1:**
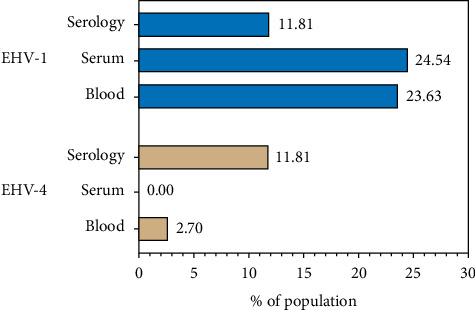
Distribution of positive samples for EHV-1 and EHV-4 in the horse population. Data are expressed in percentage.

The seroprevalence of EHV-1/4 was 11.8% (13/110), corresponding to 11 mares and 2 stallions. Regarding molecular diagnosis, EHV-1 showed an overall prevalence of 43.36% and it was nucleic acid detected in 51 horses in at least one sample (23.63% in blood, 24.54% in serum, and 7.14% in semen, corresponding to 3/42 males; Table 2; Figure 1). Noteworthy, just one sample showed positive results in PCR for whole blood and serum as well as ELISA analysis. In the case EHV-4, 3 whole blood samples were positive corresponding to a prevalence of 2.72%. In addition, ORF30 and ORF68 were detected in almost all positive samples; however, the quality of the sequences allowed to confirm the presence of EHV but not for any further bioinformatic or phylogenetic analysis.

### 3.3. Risk Factors

Positive detection rate for EHV-1/4 DNA in horses was not significantly correlated with sex (mare, OR = 0.66, *p = *0.41; stallion, OR = 1.51; *p* = 0.41), age (young, OR = 1.36, *p = *0.41; adult, OR = 0.98, *p* = 1; old, OR = 0.76, *p* = 0.74), number of births (OR = 0.39, *p = *0.35) and abortions in mares (OR = 0.25, *p = *0.20), or natural breeding (OR = 1.71, *p = *0.56) and artificial insemination methods in stallions (NM, OR = 3.88, *p* = 0.10; SC, OR = 0.53, *p = *0.54).

## 4. Discussion

Nine herpesviruses can infect equids with EHV-1 and EHV-4 with high prevalence and mortality, mainly EHV-1 [19]. EHV-1 and EHV-4 are considered different virus species by analysis of the nucleotide sequence and restriction endonucleases; nevertheless, they shared high homology at amino acid level [20]. Seroprevalence of EHV-1/-4 found in this study (11.8%) agrees with reports from El Brini et al. [21], in unvaccinated horses, and Dunowska et al. [22], in Australia. In Colombia, seroreactivity reported in horses ranging from 12% to 33% for EHV-1 and from 88% to 98.7% for EHV-4 [9, 23, 24]. Likewise, prevalence values have been reported of 65.9% in Ethiopia [25], 64% in Egypt [26], 60% in Poland [27], 53.9% in Spain [28], 48.59% in Perú [29], and 51.8% in Turkey [30] which are higher than reported in our study. In addition, studies in wild equids (zebra) also showed higher seroreactivity (84%) to EHV [31].

According to several studies, serological results are not indicative of EHV-1 infection state, since antibodies are not produced in latency, and 54%–88% of EHV-1 infections are latent in horses [9, 20, 32]. In our study, a seroprevalence of EHV-1/-4 was lower than molecular prevalence. Moreover, sometimes, samples collection can occur in the first days of lytic infection where detectable antibody response did not develop [9], as well as some do not discriminate between EHV-1 and EHV-4 [10]. Therefore, hnPCR is a likely indicator for the detection of latent and active EHV-1 in horses [23].

Furthermore, for EHV-1 detection, different targets such as glycoprotein B, glycoprotein C, and glycoprotein D genes have been used; however, these genes have a high homology with EHV-4 [11]. To avoid false positives to EHV-1, in the current study, *gH* gene was amplified, which is highly specific for EHV-1 [23]. Furthermore, the fact that blood samples were positive for PCR is indicative of viremia and thus suggestive of active infection by the viruses in the horses [33].

Molecular prevalence of EHV-1 obtained from the serum (24.54%) and blood (23.63%) samples is close to regional prevalence reported in Colombia, e.g., Orinoquía (12.7%), Cundinamarca (12%), Meta (33.7%), Antioquia (18.8%–27.8%) [9, 11, 23, 24], and similar to the reports in countries such as Uruguay (28%) [34], Iran (8%–18%) [33], Serbia (48%) [35], Egypt (36.25%) [36], Turkey (29%) [37], and New Zealand (32.7%) [22]. Recently, a prevalence of 3.4% was reported in horses with respiratory diseases in Ethiopia [38], 3% in Australia [39], and 3.55%–12% in South Korea [14, 40], which are lower than that obtained for Ibagué, Tolima. In the case of EHV-4, a prevalence in our study (2.72%) agrees with Stasiak and Dunowska, and Rola [41], who reported 0.4% by PCR assay; Radalj et al. [35], with 0.89% by multiplex nested PCR; Pusterla et al. [42], with 1.26% by qPCR; and Castro and Arbiza [34], with 6% by PCR. However, studies in Egypt showed a prevalence of EHV-4 of 30% [36], 17.4% in Turkey [37], and 18% in Iran [33]. Our results differ from the common pattern observed in Colombia and worldwide where EHV-4 prevalence is higher than EHV-1.

Recently, Pusterla et al. [43], through a systematic review, described that the sensitivity of PCR-based methods matched or exceeded to virus isolation techniques. EHV-1 has been detected in respiratory tract cells, nasal swab, serum, lymph node tissue, peripheral blood mononuclear cells, semen, and trigeminal ganglia [1, 14]. In the present study, whole blood, serum, and semen samples were used, which need less invasive protocols and avoid animal stress. Based on these samples for EHV-1 detection, a high prevalence (43.36%) was obtained, which is similar to previous studies using molecular methods in trigeminal ganglia (58%), lymph nodes (54%), tissue (81%), and nasal swab (45.1%) [11, 20, 44]. This suggests that the use of blood and serum for EHV-1 detection may represent a highly sensitive and minimally invasive method. It should be noted that nasal swabs have been reported as the one with the highest chance of EHV-1 detection mainly in early stages of infection; nevertheless, within several days, nasal shedding and viremia can occur concurrently [36, 43]. In the case of blood, it has been reported as a suitable sample in abortion outbreaks to detect EHV-1 [45]. In contrast, EHV-4 lytic infection is restricted to cells of the upper respiratory tract and leukocyte-associated viremia is extremely rare [46], which may have limited the detection in the samples of our study.

EHV-1 was simultaneously detected in blood and serum in only 2 horses, because EHV-1 detection in blood is associated with viremia and involved in the spread and transmission of the virus to endothelial cells of other organ systems [9, 23]. Otherwise, EHV-1 presence in serum may be linked to virus reactivation, since this virus quickly binds to the outer platelet membrane to induce granulosa α secretion and microvesiculation, leading to the innate immune response activation, and therefore the infection spread [47]. However, latency transcripts were not evaluated in the current study.

In agreement with other studies, EHV-1 DNA was present in the semen of three horses [48–50] suggesting the possibility of venereal transmission to mares, which can affect fertility or cause abortions [50]. However, the detection of viral DNA in semen does not predict the ability of the virus to infect mares [19]; therefore, additional studies are required to verify EHV-1 virulence in the semen.

In our study, we were unable to detect ORF30 in 10 EHV-1-positive samples and ORF68 was not detected in any positive sample, probably because the ORF copy number was lower than the PCR detection sensitivity. In addition, sequence quality allowed to confirm the EHV diagnosis, but it was not enough for further analysis.

Present study reports EHV-1 presence in blood, serum, and semen of clinically healthy horses. According to previous observation, EHV-1 was detected from horses lacking evident clinical signs [14]. Age, breed, sex, vaccination status, long-distance transportation, corticosteroids administration, hospitalization, coinfection, and stress have been reported to be possible risk factors associated with EHV-1 presence in equines [4, 14, 51].

Consistent with results, no statistical associations have been reported between age and sex and EHV-1 presence [9, 11, 21, 24]. Although not significantly different, a higher prevalence of EHV-1 was recorded in stallions (*OR* = 1.51) and in young horses (*OR* = 1.36). Similar to what was reported by Negussie et al. [38], the prevalence in young horses was higher than that of the other age groups but did not obtain statistical differences in this regard. This is in line with what has been reported in other studies, where young equines are at greater risk of developing EHV infections, since mares infected with latent EHV-1 serve as a continuous source of viral exposure to foals by horizontal transmission [1, 4, 32, 38]. In contrast, El Brini et al. [21] showed that the incidence of EHV-1 antibodies increases significantly with age.

Furthermore, the high prevalence of EHV-1 reported for Ibagué, Tolima, may be due to the immune and nutritional status of the horses [11, 52] as well as equines extensive exploitation in the municipality, since that a reduced farming and extensive exploitation can decrease viral infection transmission [11].

## 5. Conclusion

This study determined a high prevalence of EHV-1 in horses from Ibagué, Tolima, with a lower prevalence for EHV-4. Furthermore, the possibility of finding the infectious agent in whole blood, serum, and semen was demonstrated. The use of whole blood and serum samples may be useful to increase the EHV-1 detection in equines. Venereal transmission of EHV-1 through semen is suggested; however, more studies are required to demonstrate the feasibility of infection in this transmission.

## Figures and Tables

**Table 1:** Primers sequences features for EHV-1 and EHV-4 detection.

Gene	Primer	Length (bp)	Tm (°C)	Amplicon size (bp)	Reference
*ACTB*	F: TTCGAGACCTTCAACACCCCTGC	23	64.8	511	This study
	R: AGAGGTCCTTGCGGATGTCGAC	23	64.2		

*gH*EHV-1	F: AAGAGGAGCACGTGTTGGAT	20	59.3	636	[16]
	R: TTGAAGGACGAATAGGACGC	20	57.7		
	hnR: AGTAGGTCAGGCCGATGCTT	20	60.9	287	

ORF30 EHV-1	F: GCGCTACTTCTGAAAACG	18	54.3	654	[17]
	R: CCACAAACTTGATAAACACG	20	53.1		
*gB* EHV-4	F: CTGCTGTCATTATGCAGGGA	20		509	
	R: CGTCTTCTCGAAGACGGGTA	20			
	hnR: CGCTAGTGTCATCATCGTCG	20		323	
ORF68 EHV-4	F: AGCATTGCCAAACAGTTCC	19		∼600	
	R: AAGAAACCACTGCTCAACC	19			

*Note:* F: forward primer, R: reverse primer, hnR: reverse primer for heminested PCR.

**Table 2:** PCR and serology positive results for detection of EHV-1 and EHV-4 in different samples.

Sex	Age (years)	PCR						
		Whole blood		Serum		Semen		Serology
		EHV-1	EHV-4	EHV-1	EHV-4	EHV-1	EHV-4	EHV-1/-4
Female	12	−	−	+	−			−
Female	8	+⁣^∗^	−	−	−			−
Female	7	+⁣^∗^	−	−	−			−
Female	5	+⁣^∗^	−	−	−			−
Female	4	+⁣^∗^	−	−	−			−
Female	7	+⁣^∗^	−	−	−			−
Female	8	+⁣^∗^	−	−	−			−
Female	7	−	−	−	−			+
Female	6	−	−	−	−			+
Female	7	+⁣^∗^	−	+⁣^∗^	−			−
Female	12	−	−	+⁣^∗^	−			−
Female	2.5	−	−	+	−			−
Female	7	−	−	+⁣^∗^	−			−
Female	6	+⁣^∗^	−	+⁣^∗^	−			+
Female	3	+⁣^∗^	−	−	−			−
Female	5	+⁣^∗^	−	−	−			−
Female	7	+⁣^∗^	−	−	−			−
Female	10	+⁣^∗^	−	−	−			+
Female	7	−	−	−	−			+
Female	10	−	−	−	−			+
Female	6	−	−	+⁣^∗^	−			+
Female	7	−	−	+⁣^∗^	−			−
Female	8	−	−	+	−			+
Female	6	−	−	−	−			+
Female	5	−	−	+	−			−
Female	3	−	−	−	−			+
Female	7	−	−	−	−			+
Female	7	−	−	+⁣^∗^	−			−
Female	3.5	−	+	−	−			−
Female	6	−	−	+	−			−
Female	4	−	−	+⁣^∗^	−			−
Female	9	−	+	+⁣^∗^	−			−
Female	4	−	+	−	−			−
Female	10	−	−	+⁣^∗^	−			−
Female	13	−	−	+	−			−
Female	8	−	−	+	−			
Female	7	−	−	+⁣^∗^	−			−
Female	7	−	−	+⁣^∗^	−			−
Male	3	+⁣^∗^	−	−	−	−	−	−
Male	6	+⁣^∗^	−	−	−	−	−	−
Male	3	+⁣^∗^	−	−	−	−	−	−
Male	5	+⁣^∗^	−	−	−	−	−	−
Male	12	+⁣^∗^	−	−	−	+	−	−
Male	8	+⁣^∗^	−	−	−	−	−	−
Male	4	+⁣^∗^	−	−	−	+⁣^∗^	−	−
Male	6	+⁣^∗^	−	−	−	−	−	−
Male	5	+⁣^∗^	−	−	−	−	−	−
Male	5	+⁣^∗^	−	−	−	−	−	−
Male	9	+⁣^∗^	−	−	−	−	−	−
Male	7	−	−	+⁣^∗^	−	−	−	−
Male	6	−	−	−	−	+⁣^∗^	−	−
Male	8	−	−	+	−	−	−	−
Male	15	−	−	+⁣^∗^	−	−	−	−
Male	20	−	−	+⁣^∗^	−	−	−	−
Male	10	−	−	+⁣^∗^	−	−	−	−
Male	5	+⁣^∗^	−	−	−	−	−	−
Male	4	+⁣^∗^	−	−	−	−	−	−
Male	3	+⁣^∗^	−	−	−	−	−	+
Male	5	−	−	+⁣^∗^	−	−	−	−
Male	12	−	−	+⁣^∗^	−	−	−	+
Male	5	−	−	+	−	−	−	−

*Note:* Sample in red color corresponds to the only positive by PCR and serology tests simultaneously.

⁣^∗^ORF was detected.

## Data Availability

Data are available on reasonable request due to privacy/ethical restrictions.
